# Mode of Delivery Is Associated with Postpartum Depression: Do Women with and without Depression History Exhibit a Difference?

**DOI:** 10.3390/healthcare10071308

**Published:** 2022-07-14

**Authors:** Tsai-Ching Liu, Hui-Chun Peng, Conmin Chen, Chin-Shyan Chen

**Affiliations:** 1 Department of Public Finance, National Taipei University, New Taipei City 23741, Taiwan; tching@mail.ntpu.edu.tw (T.-C.L.); hcpeng@mail.ntpu.edu.tw (H.-C.P.); 2 School of Medicine, Tzu Chi University, Hualien 97004, Taiwan; 107311116@gms.tcu.edu.tw; 3 Department of Economics, National Taipei University, New Taipei City 23741, Taiwan

**Keywords:** postpartum depression (PPD), mode of delivery, elective CS

## Abstract

Whether the mode of birth delivery correlates with the incidence of postpartum depression (PPD) is still under debate. This study seeks to clarify such a correlation and to further investigate if there are any differences in the likelihood of PPD medical care use among women with and without a history of depression. A logistic regression at two assessment points (6-month and 12-month postpartum) on the National Health Insurance Database in Taiwan is performed. In total, 32,729 women were included who gave first birth from 2007 to 2011 via cesarean section (CS), elective CS, and vaginal delivery, of whom 3580 (10.9%) were diagnosed with a history of depression. Findings show that CS was associated with a higher likelihood of PPD doctor visits regardless of whether the women have a history of depression or not, but elective CS tended to have different impacts for these two groups of women. Mentally healthy women who experienced elective CS had 1.36- and 1.64-times higher risk of PDD medical care use than those who delivered vaginally, whereas previous depressive women undertaking an elective cesarean birth had no significant difference observed in incidences. A notably higher risk of elective CS delivery versus vaginal delivery for mentally healthy women suggests that elective CS is not clinically appropriate, yet it might be an alternative to vaginal delivery with careful counseling for pregnant women who experience a history of depression.

## 1. Introduction

Approximately 85% of mothers experience mental disturbances following childbirth, with baby blues and postpartum depression (PPD) being the most common mental disorders that severely affect their health [1]. A number of empirical studies provide evidence that psychosocial and biological factors are the etiological mechanisms for PPD, whose symptoms usually include mood swings, sadness and crying, weight and appetite changes, a lack of interest in daily activities, insomnia or hypersomnia, serious concerns about their child, fatigue or loss of energy, feelings of worthlessness, or excessive or inappropriate guilt and thoughts of death. More importantly, obsessive thoughts about harming their children or themselves are also some of the more characteristic symptoms of PPD, which may further worsen the mother’s mental state condition in a sort of a “vicious circle” mechanism. In general, PPD is highly associated with the risk of maternal suicide and could be a more common cause of peripartum mortality than postpartum hemorrhage or hypertensive disorders [2].

The risk factors of PPD are complicated and are defined in terms of a previous history of depression and anxiety [3,4], postpartum obstetric complications [5], maternal age [6,7], hormonal imbalances (e.g., oxytocin and estrogen) [8], lack of familial support [9], unstable economic status [10,11,12,13], and food intake patterns [14,15,16]. The results of these studies indicated that mode of delivery, postpartum complications, maternal age, and a prior history of depression increase PPD risk. While the issue over whether or not the mode of delivery affects women’s postpartum psychological well-being has received considerable attention, the association between mode of delivery and PPD is still unclear and controversial.

The cesarean section (CS) rate in the past few decades has dramatically increased worldwide both in many developed and developing countries, raising concerns among public health researchers. Some previous studies have provided evidence showing that there are differences in the incidences of PPD between CS and vaginal delivery and confirmed significantly higher incidences of PPD in women who delivered by CS [4,17,18,19,20,21,22,23,24,25]. On the contrary, Chaaya et al. [26] found that women who undergo vaginal delivery have higher PPD symptoms than those who have a cesarean section.

A plethora of studies has revealed no evidence that cesarean delivery has a higher chance of causing PPD than vaginal delivery does. Women’s postpartum mental health disturbance was not found to be associated with mode of delivery [27,28,29,30,31,32]. Olieman et al. [31] argued that women who requested elective CS do not seem to have a higher probability of PPD incidence than women who delivered vaginally; however, women who preferred elective CS, but were forced to deliver vaginally, might have a higher incidence of PPD than women who delivered vaginally without any preference. The factor affecting the risk of PPD is concluded to be the preference of mode of delivery rather than the actual mode of delivery per se.

A history of mental health problems puts women at risk for depression in the postpartum period. Several studies have shown that a history of depression may trigger PPD and is one of the predominant factors for the risk of PPD. For example, Silverman et al. [4] showed that the risk of PPD for women with a history of depression is more than 20 times higher than for women without such a history. Among women with no history of depression, CS does raise the risk of PPD. Conversely, Eckerdal et al. [5] confirmed that mode of delivery has no direct effect on PPD, but a negative delivery experience mediates an indirect association between clinical CS and PPD. Women who had antenatal psychological distress and underwent CS delivery may be regarded as a target for monitoring PPD [33]. Since a history of depression is proven to be correlated with postpartum mental health, it is logical to consider the history of depression as an important risk factor when further investigating the relationship between mode of delivery and PPD.

Because Taiwanese women are increasingly pursuing higher educational levels and professional positions, they tend to delay marriage, which results in putting off pregnancy. Strelow et al. [7] suggested that maternal age is a strong risk factor associated with PPD, but according to findings in previous studies, the impact of maternal age on PPD remains inconclusive. Davé et al. [34] showed that the incidence of PPD is higher when women are younger or older, but Muraca et al. [35] reported that the incidence of PPD increases with maternal age. Focusing on women with a history of depression, studies have suggested that a history of depression increases the risk of depression during the postpartum period, and this risk increases with maternal age [4,6]. In sum, maternal age is viewed as an important factor, but its impact on PPD is still in dispute. Thus, we consider maternal age as a risk factor in this study.

Taiwan is a country with one of the highest CS rates at above 30%, far surpassing the acceptable rate of 15% as suggested by the World Health Organization. Many studies undertaken in Taiwan have assessed the impact of the mode of delivery on the physical health of the mothers and infants and found that CS causes more postpartum maternal care use [36,37]. Chen et al. [38] explored the association between the choice of birth delivery and maternal psychiatric health and suggested that no relationship exists. As far as we know, most existing studies investigated the association between PPD and mode of delivery via comparing women who underwent CS and vaginal delivery. Research on the incidences of PPD among the subtype of delivery mode (i.e., vaginal delivery vs. elective CS) is quite limited.

One advantage of this study is being able to provide a clear distinction among delivery modes, from vaginal delivery to elective CS and CS. Moreover, the majority of the above studies only analyzed the association between mode of delivery and PPD in a specific postpartum period. The data used herein provide an exclusive opportunity to estimate the relationship over different postpartum periods, covering 6 months and 12 months postpartum. Therefore, the objective of this study is to assess whether CS (elective CS) is associated with a higher risk of PPD and to examine if there are any differences by CS (elective CS) during the 6-month and 12-month postpartum periods between two groups of women with and without a history of depression. Since one of the main aims is to understand the relationship between PPD and maternal self-chosen delivery, we investigate elective CS and vaginal delivery. We do not report modelling data on clinical CS as this cannot be planned or chosen by oneself.

## 2. Materials and Methods

### 2.1. Data

The dataset was selected from the 2010 Longitudinal National Health Insurance Database (2010LNHID) in Taiwan. Under NHI, all contracted medical institutions must submit claims electronically for reimbursement. With this compulsory feature, approximately 99% of people in Taiwan are covered by NHI, making the sample very representative of the total population and conveniently used worldwide. The dataset contains detailed information on the services provided, the diagnosis (ICD-9 code), the date and duration of each service, total expenditure of each visit, and the age and gender of the beneficiary.

We identified women who gave birth in 2007–2011 as our study sample via diagnosis-related group (DRG) codes and classified the observations into three groups—vaginal delivery (DRG = 0373A), elective CS (DGR = 373B), and clinical CS (DRG = 371A)—to compare how mode of delivery causes differences in PPD medical care utilization. Using the unique identifier of each beneficiary, we further linked their records of PPD medical care utilization prior to their deliveries and all PPD medical claims during the postpartum period of the first 6 and 12 months after their deliveries.

From the dataset of outpatient care use, we are able to identify whether mothers have a history of depression before birth and PPD medical care use after birth for each woman based on the ICD-9 code. We define women as having a history of depression if they have a diagnosis of ICD-9-CM 296-311, 648.40, 648.42, and 648.44 based on the previous studies by Silverman et al. [4] and Savitz et al. [39]. They included diagnoses of PPD, major depressive disorder (unipolar), unspecified episodic mood disorder, or depressive disorder in the period of 12-month postpartum for PPD according to American College of Obstetrics and Gynecology guidelines and the Agency for Healthcare Research and Quality [40]. It is noteworthy that the 12-month timeline for PPD may exhibit controversy according to the American Psychiatric Association [41], which classified peripartum depression as a major depressive disorder during pregnancy or within four weeks postpartum, while the World Health Organization defined PPD six weeks after delivery [42]. To be consistent with the studies indicated above, we define PPD as a depression diagnosis in the period of 12 months postpartum. In terms of the definition of a history of depression being consistent with Silverman et al. [4], we classify depression history as a clinical diagnosis of depression any time prior to the date of delivery using the same ICD-9 code.

Among women with vaginal deliveries, undergoing an instrument-assisted one could increase the risk of PPD, and so instrument-assisted vaginal deliveries (forceps or vacuum-assisted vaginal deliveries, ICD-9-CM 6695) are also excluded. As diabetes (ICD-9-CM 250,648.8) and hypertension (ICD-9-CM 401-405,642) could lead to higher risks of PPD incidence, these two health conditions will be controlled in the analysis. After deleting missing values and mothers experiencing a miscarriage (ICD-9-CM 634-639), multiple birth (ICD-9-CM 651), and non-first-time pregnancy, the final sample consists of 29,149 pregnant women without prior depression and 3580 pregnant women with prior depression during the study period. Vaginal deliveries, CS, and elective CS ended up with sample sizes of 18,869, 10,280, and 643, as well as 2080, 1500, and 109 observations for the two groups, respectively, as shown in Table 1.

### 2.2. Empirical Model

This study undertakes two models, each with two estimations (CS vs. vaginal and elective CS vs. vaginal) at two assessment points—6-month and 12-month postpartum periods—in order to explore whether or not an impact of delivery mode on the probability of PPD medical care utilization differs for these two postpartum periods. Model I, as indicated in Table 2, estimates the impact for women with no history of depression and contains two estimations. In the first estimation (CS vs. vaginal), the total number of observations was 29,149, including 10,280 and 18,869 for women with no history of depression undertaking CS and vaginal delivery, respectively. In the second estimation, there were 19,512 observations, which were 643 and 18,869 for elective CS and vaginal delivery, respectively. Model II explores whether women with a history of depression undergoing CS or elective CS experience a higher risk of PPD. As shown in Table 3, there were only 3580 and 2189 observations in the first and second estimations, respectively. In the first estimation (CS vs. vaginal), 1500 and 2080 women with a history of depression undertook CS and vaginal delivery, respectively, while there were only 109 for elective CS and 2080 for vaginal delivery in the second estimation (elective CS vs. vaginal).

We adopt the logistic regression and estimate the odds ratio of the probability of PPD doctor visits during the post-delivery period, as the means for comparing differences in the prevalence of PPD medical care utilization between women who underwent vaginal deliveries and those who underwent surgical procedures. The endogenous variable takes 2 values: 0 if the women did not have any PPD doctor visits, or 1 if the women had at least one visit. Other essential variables, such as maternal age, geographic characteristics, hospital levels, diabetes, and hypertension are also taken into account in the estimations. All statistical analyses are performed using the STATA version 16.0 statistical software package.

## 3. Results

### 3.1. Descriptive Statistics

Table 1 shows the characteristic of the sample for women with and without a history of depression by mode of delivery. Since we conduct data collection at two assessment points (6-month and 12-month postpartum periods), descriptive statistical analyses are performed on each group. Table 1 lists the descriptive statistics.

#### 3.1.1. Women without a History of Depression

##### Mode of Delivery

Among the total sample of women without a history of depression (n = 29,149), 10,280 and 643 women undertook CS and elective CS, constituting 35.27% and 2.21%, respectively. Table 2 presents the statistics of the mode of delivery (vaginal delivery, CS, and elective CS), in which the means of incidences of PPD medical care utilization in the 6- and 12-month postpartum periods are 1.46%, and 3.06%, respectively. Women with CS or elective CS are much more prone to incur PPD medical care use at 6-month postpartum compared to vaginal deliveries (1.80% vs. 1.28%; 2.18% vs. 1.28%). CS and elective CS also present a greater probability of PPD medical care use than vaginal deliveries (3.67% vs. 2.72%; 4.04% vs. 2.72%) at 12 months postpartum.

##### Maternal Age

Among the four age groups, approximately 60% of mothers are 30 years old or older, about 40% are within the 30–34 age range, while only 10.55% are under 25 years old. Compared to the youngest mothers (1.59%), those aged 25–29 (1.50%) and those above 35 (1.53%) present a slightly lower likelihood of PPD medical care use at 6 months postpartum, while women aged 30–34 have the lowest rate (1.37%). In a similar finding for 12 months postpartum, the subgroup of women under 25 years old has the highest rate (3.58%), while mothers aged 30–34 have the lowest percentage (2.68%). In sum, regardless of the estimated point at 6 or 12 months postpartum, the majority of mothers in the 30–34 age group have the smallest incidences of PPD. This is probably due in large part to the trend of Taiwanese women delaying marriage, which may lead to delayed pregnancy.

#### 3.1.2. Women with a History of Depression

##### Mode of Delivery

In total, 3580 women with a history of depression are included in the final analysis, with 1500 (41.9%) and 109 (3.04%) delivering by CS and elective CS. The incident rate of postpartum depression is 10.03% at 6 months postpartum and 15.45% at 12 months postpartum.

As seen from Table 1, of the 359 women with postpartum depression at 6 months postpartum, 171 had CS (n = 1500), 12 had elective CS (n = 109), and 188 delivered vaginally (n = 2080). The incidences of PPD medical care utilization at 6 months postpartum for women delivering by overall CS or elective CS are higher than that of the control group (women delivering vaginally), with the respective figures at 11.40% and 11.01% versus 9.04%. Similarly, the percentage of PPD medical care utilization for women delivering by CS or elective CS during the 12-month postpartum period is also dramatically higher than those who undertook vaginal deliveries (17.60% and 20.18% vs. 13.89%).

##### Maternal Age

Among the four maternal age groups of mothers with a history of PPD, almost two-thirds of the pregnant women are within the range of above 30 years old, while the remaining groups constitute 29.16% and 10.31% for the subgroups of ages 25–29 and below 24, respectively. Compared with the youngest pregnant women, those aged 25–29, 30–34, and above 35 years more frequently requested PPD doctor visits at 6 months postpartum (6.5% vs. 9.1%, 9.13% and 14.72%, respectively). Similarly, the risk of PPD medical care use at 12 months postpartum for women with a history of depression is also found to increase with advancing maternal age, which is 12.47%, 13.31%, 14.93%, and 20.82% for those aged under 24, 25–29, 30–34, and above 35 years old, respectively.

### 3.2. Empirical Results

#### 3.2.1. Women without a History of Depression

##### Mode of Delivery

As Table 2 indicates, the coefficient of cesarean section is statistically significant and positive in the 6-month and 12-month postpartum periods at OR = 1.398 and 1.365 (*p* < 0.001), respectively. This result implies that women who undertake cesarean section are 1.398 and 1.365 times more likely to incur PPD outpatient care use after 6- and 12-month postpartum periods than those with vaginal deliveries. In a comparison between elective CS and vaginal delivery, the coefficient of elective CS is also statistically significant and positive in the 6- and 12-month postpartum periods (OR = 1.876 and 1.648: *p* < 0.05). This finding suggests that women without a history of PPD who undergo elective CS are 1.876 and 1.648 times more likely to incur PPD than those with vaginal delivery in the 6-month and 12-month postpartum periods.

##### Maternal Age

Four of all the age dummies are, as expected, found to have a negative coefficient. Younger women aged 25 to 29 years old have a higher risk of PPD compared with mothers aged 24 and below. Women over the age of 30–34 years have a statistically significant increased risk of PPD at 12 months postpartum. Women over age 35 are considered to be advanced and surprisingly are not found to have a statistically significant increased risk of PPD medical care use at 6- or 12-month postpartum periods.

#### 3.2.2. Women with a History of Depression

Mode of delivery. Delivery mode (CS vs. vaginal delivery) has a statistically significant and positive coefficient at 12 months postpartum with OR = 1.212 (*p* < 0.05). Regarding elective CS versus vaginal delivery, no apparent differences appear in their impact at either 6 or 12 months postpartum (Table 3).

Maternal age. All age dummy variables are found to have a positive coefficient, but only the subgroup of age 35 years old and above reaches a statistically significant level. In comparison between CS and vaginal delivery, the odds ratio of age 35 and above is 2.559 and 1.894 at 6 and 12 months postpartum, respectively. This finding implies that older women, aged 35 and above, have a higher risk of PPD medical care use compared with mothers aged below 25. Similarly, in the estimation of elective CS versus vaginal delivery, the variable of age 35 years old and above is found to have an odds ratio of 2.554 (*p* < 0.01) and 1.765 (*p* < 0.05) at two estimated time points, suggesting that mothers with a history of depression who are aged 35 and above are 2.55 and 1.77 times more likely to experience PPD medical care use.

## 4. Discussion

There have been few studies that have quantitatively assessed incidences of PPD medical care utilization by CS (elective CS) versus vaginal delivery procedures, particularly so for making a comparison between mentally healthy women and depressive women. The aim of this study is to explore the association between delivery modes (CS vs. vaginal delivery; elective CS vs. vaginal delivery) in the 6-month and 12-month post-delivery periods among women with and without a history of depression before delivery. The analysis is restricted to women diagnosed with depression within 1 year after birth.

The statistics show that the incidence rate of PPD doctor visits at 6 and 12 months postpartum is 1.46% and 3.06% for women without a history of depression, whereas it is 10.03% and 15.45% for those with a history, making it comparable to the prevalence of PPD at 10–15% as reported by international studies [6]. Among women without a history of depression, the average percentage of PPD outpatient care utilization at two estimation points for CS (elective CS) is much higher than that for vaginal delivery at 1.80% (2.18%) and 3.67% (4.04%) versus 1.28% and 2.72%, respectively.

After making the empirical estimations, the results show that CS (elective CS) has a significantly increased risk of PPD compared to a vaginal delivery with an odds ratio of 1.398 (1.365) and 1.876 (1.648). This finding indicates that the risk of PPD medical care use is associated with CS even with elective CS at 6- and 12-month postpartum periods, which is 1.398 (1.365) and 1.876 (1.648) times higher than vaginal delivery among mentally healthy women. This finding is consistent with previous studies [4,23,37].

A similar trend appears in women with a history of depression, with the mean risk of PPD doctor visits at these two estimated points by CS (elective CS) higher than those for vaginal delivery; it is 11.40% (11.01%) and 17.60% (20.18%) versus 9.04% and 13.89%. The empirical results show that the incidence of PPD outpatient care use by CS is statistically significant at 12 months postpartum with an odds ratio of 1.212, but there is no difference observed in the risk for elective CS. This finding suggests that compared to vaginal delivery, CS increases the probability of PPD medical care use for previously depressive women by 1.212 times. Consistent with the evidence of recent studies [4,23,43], our results show that women who underwent CS (vs. vaginal delivery) tend to have a higher probability of PDD; however, elective CS tends to increase the risk only for women who have no history of depression.

Strelow et al. [7] pointed out that of the many risk factors associated with PPD, maternal age seems to be a strong one. The incidence of PPD increases in both younger and older women. Another study by Petrosyan et al. [6] indicated that the risk of PPD is 1.19 times higher among women younger than 25 years old compared to those aged over 25 years. In this study, maternal age appears to have an inconsistent impact for these two groups of women with and without a history of depression. Among women who have a history of depression, the likelihood of PPD doctor visits increases with advancing age, while surprisingly, younger women tend to incur PPD in the population without a history of depression. Previously depressive women as old as age 35 and above are approximately 2.6 times more likely to incur a PPD doctor visit than those aged under 25. However, mentally healthy women aged 30–34 are around 0.7 times less likely to seek a PPD doctor visit than those aged under 25.

Another confounder is that the geographic region of central Taiwan is statistically significant with a positive coefficient, implying that geographic location is an important driver of PPD incidence. This finding suggests that among women without a history of depression, those living in the central region who received CS or elective CS were more likely to develop postpartum depression at 6 and 12 months postpartum than those in the northern region. A possible explanation is that the protective effect of adequate prenatal counseling reduces the risk of PPD in the mother. Women in the central region use less antenatal care than women in the northern region, which may in turn increase the likelihood of developing PPD. This is partly consistent with the findings of a recent study in Taiwan by Liu et al. [44], who provided evidence that women in central Taiwan are less likely to receive adequate prenatal care, which could lead to more PPD outpatient care use. There is still an uneven distribution of medical resources in Taiwan, especially preventive care, even after the implementation of National Health Insurance. However, we do not observe a significant effect among women who have a history of depression. The discrepancy of impact between the two groups of mothers implies that regional disparity in the incidence of PPD only exists for women without a history of depression who undertook CS or elective CS. This underlines the importance of distinguishing the mode of delivery in studying the effect of geographic location on the risk of PPD for mentally healthy women.

In sum, compared to vaginal delivery, mentally healthy women who experienced CS tended to have 1.398 and 1.8 times higher risk of PPD medical care use after 6 and 12 months following birth. Mentally healthy women who have elective CS have 1.365 and 1.648 times higher risk of PPD medical care use than those who deliver vaginally. This finding implies that women without a history of depression undergoing CS regardless of types have higher odds of PPD medical care utilization during their first year postpartum. In contrast to women without a history of depression, previously depressive women undertaking an elective cesarean birth exhibit no significantly different prevalence of PPD doctor visits as compared to those with vaginal delivery. This finding is consistent with evidence provided by Olieman et al. [31], Silverman et al. [4], Eckerdal et al. [5], and Smithson et al. [24], who demonstrated that elective CS does not have a higher risk of PPD. Smithson et al. found that elective CS does not correlate with PPD risk in the immediate postpartum period. Silverman et al. [4] are one of the few studies classifying pregnant women into two groups with or without a history of depression. No apparent relationship between elective and PPD doctor visits was observed for the group of women with depression. Interestingly, our finding is somewhat different from the evidence by Henderson and Quenby [33], who concluded that women with depression following elective CS have a lower risk of PPD.

This study has many strengths. First, with the establishment of Taiwan’s NHI in 1995 with extremely low copayments, the health care system in the country has become easily accessible and financially affordable for all women. This equal accessibility has reduced the likelihood of selection bias that may result from discrepancies in socioeconomic status or geographic location. Second, we are able to gather data on the diagnosis of PPD history as NHI covers every illness treatment of all Taiwanese people (approximately 23 million), with records comprising outpatient care, emergency care, and inpatient care. Third, offering comprehensive, affordable, and accessible health care provided by the NHI Program, the health care system in Taiwan has enabled women with mild symptoms of psychological problems to seek immediate medical care. Consequently, socioeconomic status is not a factor that differentiates the identification of PPD medical care utilization. Finally, the adoption of NHI claims data helps avoid recall bias, which is typically an issue with self-reported survey data.

There are some limitations that need to be addressed. First, our data are claims in nature and lack demographic characteristics, causing us to be unable to fully explain the variation in PPD medical care utilization. The LNHID database provides the best nationally representative information in Taiwan, thus allowing us to answer our research question, particularly given its accurate account of the probability of PPD outpatient health services. Second, some potential factors found to be associated with the incidences of PPD in earlier studies are not able to be controlled for explicitly herein. For example, the database provides little information on fetus characteristics (premature, birthweight, gestational age, gender, parity, etc.), maternal obstetric history, (previous pregnancies and maternal BMI), and socioeconomic status (education and income) for the mothers or their households. Third, elective CS is compared to vaginal delivery in this study, but there is no distinction that can be made between normal vaginal delivery and forced vaginal delivery. Since forced vaginal delivery induces an important risk factor for PPD, a deeper exploration of this issue in a future study would be helpful. Fourth, although the dataset in this study, 2010LNHID, indeed has a certain lag, it still provides some valuable results. Using a more recent dataset to investigate the association with mode of delivery and PPD is a direction for future study.

## 5. Conclusions

This study does confirm that CS is a risk factor of PPD for both mothers with and without a history of depression. Elective CS is also identified as an increasing risk of PPD only for women without any previous mental health problems. A notably higher risk of elective CS than for vaginal delivery suggests that elective CS is clinically inappropriate for mentally healthy women when choosing how to give birth; however, elective cesarean delivery is not associated with a higher risk of PPD for women who experience a history of depression during pregnancy, as compared to vaginal delivery. This should be taken into account when previously depressive women request an elective CS as an alternative to vaginal delivery with careful counseling.

## Figures and Tables

**Table 1:** Characteristic of the sample for women with and without a history of depression.

Characteristic	No History of Depression						History of Depression					
	Total Sample		6 Months		12 Months		Total Sample		6 Months		12 Months	
	No.	%	No.	%	No.	%	No.	%	No.	%	No.	%
Variable	29,149	100.00	427	1.46	891	3.06	3580	100.00	359	10.03	553	15.45
Mode of Delivery												
Vaginal Delivery	18,869	64.73	242	1.28	514	2.72	2080	58.10	188	9.04	289	13.89
C-section (CS)	10,280	35.27	185	1.80	377	3.67	1500	41.90	171	11.40	264	17.60
Elective CS	643	2.21	14	2.18	26	4.04	109	3.04	12	11.01	22	20.18
Maternal Age												
24 and under	3076	10.55	49	1.59	110	3.58	369	10.31	24	6.50	46	12.47
25–29	9171	31.46	138	1.50	305	3.33	1044	29.16	95	9.10	139	13.31
30–34	11,621	39.87	159	1.37	311	2.68	1413	39.47	129	9.13	211	14.93
35 and above	5281	18.12	81	1.53	165	3.12	754	21.06	111	14.72	157	20.82
Hospital Level												
Medical Center	5333	18.30	57	1.07	141	2.64	598	16.70	50	8.36	75	12.54
Regional Hospital	7824	26.84	143	1.83	265	3.39	968	27.04	89	9.19	145	14.98
District Hospital	7061	24.22	102	1.44	201	2.85	898	25.08	95	10.58	135	15.03
Clinics	8931	30.64	125	1.40	284	3.18	1116	31.17	125	11.20	198	17.74
Geographic Area												
North	14,695	50.41	192	1.31	387	2.63	1670	46.65	158	9.46	249	14.91
Center	6426	22.05	123	1.91	247	3.84	878	24.53	89	10.14	143	16.29
South	7330	25.15	101	1.38	232	3.17	926	25.87	95	10.26	140	15.12
East	698	2.39	11	1.58	25	3.58	106	2.96	17	16.04	21	19.81
Comorbidities												
Diabetes												
No	26,550	91.08	373	1.40	804	3.03	3195	89.25	327	10.23	498	15.59
Yes	2599	8.92	54	2.08	87	3.35	385	10.75	32	8.31	55	14.29
Hypertension												
No	28,016	96.11	398	1.42	837	2.99	3353	93.66	328	9.78	510	15.21
Yes	1133	3.89	29	2.56	54	4.77	227	6.34	31	13.66	43	18.94

**Table 2:** Logistic Regressions for Women without History of Depression.

Variable	CS vs. Vaginal				Elective CS vs. Vaginal			
	6 Months		12 Months		6 Months		12 Months	
	Odds Ratio	95% CI	Odds Ratio	95% CI	Odds Ratio	95% CI	Odds Ratio	95% CI
Mode of Delivery								
C-section (CS)	1.398 ***	(1.149–1.700)	1.365 ***	(1.190–1.566)	1.876 *	(1.082–3.254)	1.648*	(1.101–2.469)
Maternal Age								
25–29	0.922	(0.662–1.283)	0.915	(0.732–1.144)	0.800	(0.543–1.177)	0.807	(0.622–1.048)
30–34	0.828	(0.596–1.152)	0.731 **	(0.584–0.916)	0.805	(0.549–1.181)	0.708 **	(0.544–0.921)
35 and above	0.883	(0.611–1.274)	0.824	(0.641–1.060)	0.720	(0.447–1.162)	0.727	(0.527–1.005)
Geographic Area								
Center	1.464 ***	(1.162–1.844)	1.443 ***	(1.224–1.700)	1.472 *	(1.088–1.992)	1.378 **	(1.113–1.707)
South	1.004	(0.785–1.284)	1.179	(0.997–1.395)	1.314	(0.967–1.783)	1.479 ***	(1.199–1.823)
East	1.086	(0.587–2.008)	1.309	(0.865–1.981)	1.333	(0.647–2.746)	1.446	(0.877–2.385)
Hospital Level								
Medical Center	0.782	(0.568–1.076)	0.894	(0.726–1.102)	0.718	(0.468–1.101)	0.858	(0.656–1.121)
Regional Hospital	1.296*	(1.014–1.656)	1.093	(0.920–1.298)	1.285	(0.938–1.76)	1.063	(0.853–1.325)
District Hospital	1.031	(0.790–1.345)	0.891	(0.740–1.072)	1.094	(0.783–1.528)	0.897	(0.709–1.134)
Comorbidities								
Diabetes	1.485 **	(1.109–1.990)	1.139	(0.907–1.429)	1.408	(0.948–2.091)	1.043	(0.764–1.422)
Hypertension	1.643 **	(1.128–2.393)	1.509 **	(1.139–1.998)	1.190	(0.609–2.327)	1.141	(0.706–1.844)
Constant	0.012 ***	(0.009–0.017)	0.029 ***	(0.023–0.036)	0.012 ***	(0.008–0.018)	0.030 ***	(0.023–0.039)
Observations	29,149		29,149		19,512		19,512	

* *p* < 0.05, ** *p* < 0.01, *** *p* < 0.001. Reference groups: Age 24 and under; Clinics; North.

**Table 3:** Logistic Regressions for Women with History of Depression.

Variable	CS vs. Vaginal				Elective CS vs. Vaginal			
	6 Months		12 Months		6 Months		12 Months	
	Odds Ratio	95% CI	Odds Ratio	95% CI	Odds Ratio	95% CI	Odds Ratio	95% CI
Mode of Delivery								
C-section (CS)	1.167	(0.935–1.456)	1.212 *	(1.008–1.458)	1.097	(0.586–2.055)	1.370	(0.839–2.237)
Maternal Age								
25–29	1.471	(0.921–2.347)	1.095	(0.763–1.570)	1.672	(0.946–2.956)	1.014	(0.664–1.549)
30–34	1.495	(0.945–2.363)	1.269	(0.896–1.797)	1.604	(0.911–2.825)	1.076	(0.710–1.631)
35 and above	2.559 ***	(1.599–4.094)	1.894 ***	(1.312–2.735)	2.554 **	(1.401–4.657)	1.765 *	(1.120–2.781)
Geographic Area								
Center	1.071	(0.808–1.419)	1.112	(0.884–1.401)	1.143	(0.797–1.640)	1.197	(0.888–1.614)
South	1.086	(0.828–1.424)	1.019	(0.811–1.280)	0.945	(0.644–1.387)	0.923	(0.670–1.271)
East	1.893 *	(1.085–3.302)	1.480	(0.889–2.463)	1.815	(0.922–3.570)	1.582	(0.867–2.888)
Hospital Level								
Medical Center	0.670 *	(0.471–0.951)	0.622 **	(0.463–0.836)	0.599 *	(0.373–0.961)	0.637 *	(0.430–0.943)
Regional Hospital	0.778	(0.580–1.043)	0.801	(0.631–1.019)	0.781	(0.537–1.138)	0.834	(0.611–1.139)
District Hospital	0.898	(0.675–1.194)	0.797	(0.625–1.016)	0.749	(0.505–1.110)	0.729	(0.523–1.016)
Comorbidities								
Diabetes	0.763	(0.520–1.120)	0.893	(0.656–1.215)	0.701	(0.406–1.209)	0.957	(0.634–1.443)
Hypertension	1.325	(0.887–1.981)	1.183	(0.833–1.680)	0.867	(0.425–1.769)	0.842	(0.466–1.522)
Constant	0.070 ***	(0.044–0.112)	0.148 ***	(0.103–0.212)	0.073 ***	(0.041–0.129)	0.166 ***	(0.108–0.255)
Observations	3580		3580		2189		2189	

* *p* < 0.05, ** *p* < 0.01, *** *p* < 0.001. Reference groups: Age 24 and under; Clinics; North.

## Data Availability

Not available.
